# Scatterogram: a method for outlining the body during lymphoscintigraphy without using external flood source

**DOI:** 10.2478/v10019-011-0010-y

**Published:** 2011-03-29

**Authors:** Mehdi Momennezhad, Seyed Rasoul Zakavi, Vahid Reza Dabbagh Kakhki, Ali Jangjoo, Mohammad Reza Ghavamnasiri, Ramin Sadeghi

**Affiliations:** 1 Nuclear Medicine Research Center, Mashhad University of Medical Sciences, Mashhad, Iran; 2 Surgical Oncology Research Center, Mashhad University of Medical Sciences, Mashhad, Iran; 3 Cancer Research Center, Mashhad University of Medical Sciences, Mashhad, Iran

**Keywords:** breast cancer, scattered photons, body outlining, lymphoscintigraphy, intradermal injection, body contour

## Abstract

**Background:**

We evaluated the feasibility of outlining the body with scattered photons using a low dose intradermal injection of the radiotracer.

**Patients and methods.:**

Sixty breast cancer patients were included into the study. 30 minutes post radiotracer injection static lymphoscintigraphy images were acquired using low energy high resolution collimator in anterior and lateral views. For patients with 2-day protocol another set of images was taken 20 hours post-injection. Two photopeaks were used during imaging: 1-Tc-99m (130-150 keV) and 2- Scatter photons (60-120). The fusion image of these two images was constructed by NM-NM fusion workflow of the workstation. The usual body outline of the patients was also acquired in 20 cases using the external flood source without moving the patients from their positions.

**Results:**

The early (30 minute image) scatterograms of the patients clearly showed the contour of the body. The 20 hour scatterograms were not as high quality as the corresponding early images. The constructed overlaid images showed the location of the axillary sentinel nodes and the body contours clearly for early scatterograms but not the delayed (20 hour) ones. The processing of the images for the reconstruction of overlaid scatterograms took the mean time of 10±5 seconds.

**Conclusions:**

Imaging the scattered photons is feasible for the intradermal low dose injection of the radiotracers in order to outline the body contour. This imaging method does not increase the radiation exposure of the patients or operators and does not extend the time of imaging either.

## Introduction

Sentinel lymph node biopsy is considered as the standard method for axillary staging of breast cancer patients without clinically involved axilla which can significantly decrease the morbidity of these patients.^1^,^2^ Lymphoscintigraphy with gamma cameras can show the sentinel lymph nodes and is recommended to be performed before surgery.^3^ However, plain lymphoscintigraphy images do not show any anatomical detail or exact location of the sentinel nodes in axilla which are very important factors for surgery planning.^4^ Skin pen marking^1^, stereoscopic lymphoscintigraphy^5^ and body outlining^6^ are three solutions to deal with this problem. Several methods are in use for outlining the body contour during lymphoscintigrpahy imaging with their own advantages and drawbacks.^7^ These include: (1) moving a small point source of Tc-99m around the edge of the patient manually^4^,^8^; (2) external sources of gamma rays such as Co-57 flood source^9^ and Gd-153 line source^4^; (3) imaging the scattered photons of the injected radiotracer^10^ and (4) intravenous injection of a small amount of Tc-99m-pertechnetate.^11^

Imaging the scattered photons (which we call scatterogram) is an easy way to outline the body during lymphoscintigraphy imaging without any additional radiation exposure to patients and technologists. This method has been previously described by Fujii *et al.*^10^,^12^ for breast cancer patients using peritumoral injection of 111 MBq radiotracer. We routinely use intradermal injection of 18.5-37 MBq Tc-99m-antimony sulfide colloid for sentinel lymph node mapping.^1^ In the current study we evaluated the feasibility of outlining the body with scattered photons for this protocol of sentinel lymph node biopsy.

## Patients and methods

**FIGURE 1:**
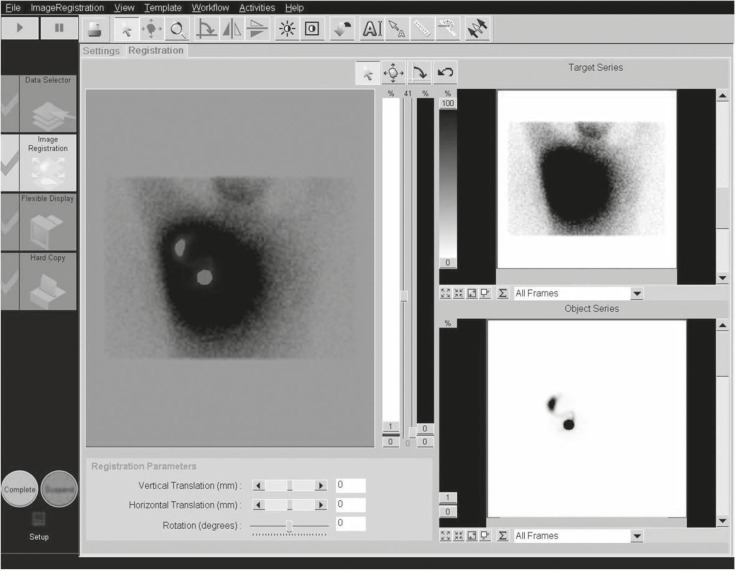
NM-NM fusion workflow. We used this workflow to construct the overlay image of the lymphoscintigraphy and scattered photons. The original images with Tc-99m photopeak and scatter photopeaks are shown on the right side of the image. The constructed overlay image is shown on the left side. Note clear visualization of the axillary sentinel node as well as the outline of the patient.

Sixty female patients with the diagnosis of breast cancer without clinically involved axilla were included into the study. The intradermal injection of Tc-99m-antimony sulfide colloid (18.5 MBq or 37 MBq for 1 day and two-days protocols respectively) was used in a peri-areolar fashion. Static lymphoscintigraphy images were acquired 30 minutes post-injection using a dual head variable angle gamma camera (E.CAM Siemens) equipped with low energy high resolution collimator in anterior and lateral views (5 minutes/image and 128×128 matrix). For patients with 2-day protocol another set of images was taken 20 hours post-injection. Two photopeaks were used during imaging: 1-Tc-99m photopeak (130–150 keV) and 2- Photopeak for scatter photons (60–120). The outputs of each photopeaks were saved as separate images in the computer. The fusion image of these two images (lymphoscintigraphy with Tc-99m photopeak and the scatterogram) was constructed by NM-NM fusion workflow of the workstation after normalizing the intensity of the images to the sentinel node activity for the Tc-99m and the corresponding area for the scatterogram images. The images were simply overlaid on each other (Figure 1). The usual body outline of the patients was also acquired in 20 cases using external flood source without moving the patients from their positions. The resulting overlaid images were compared with the usual lymphoscintigraphy as well as outline images of the patients considering the quality of body outlining and anatomical location of the sentinel node in the axilla by two nuclear medicine specialists.

After the completion of the imaging, the patients were sent to the surgery ward for usual axillary sentinel node mapping with gamma probe as well as blue dye injection.

## Results

**FIGURE 2:**
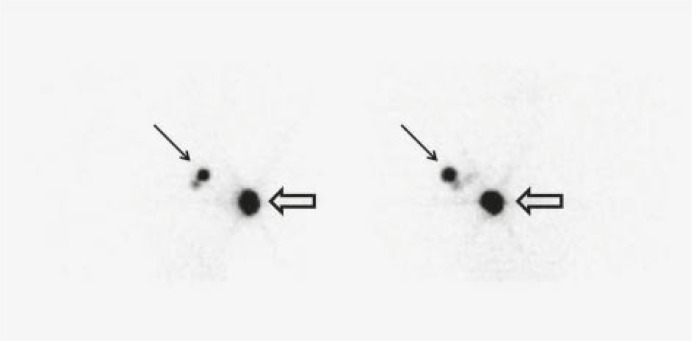
Anterior (right) and Lateral (left) lymphoscintigraphy images of a patient. Note the injection site (hollow arrows) as well as sentinel nodes (arrows).

**FIGURE 3:**
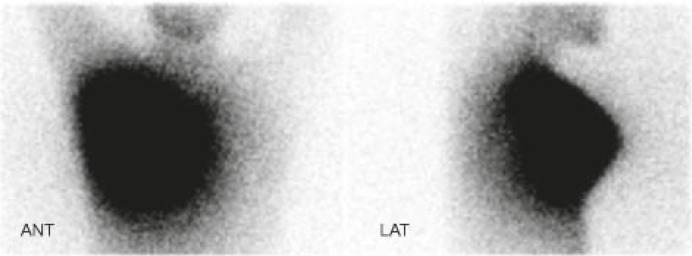
Anterior (left) and Lateral (right) scatter photon images of a patient acquired 30 minutes post-injection. Note clear visualization of body outline.

**FIGURE 4:**
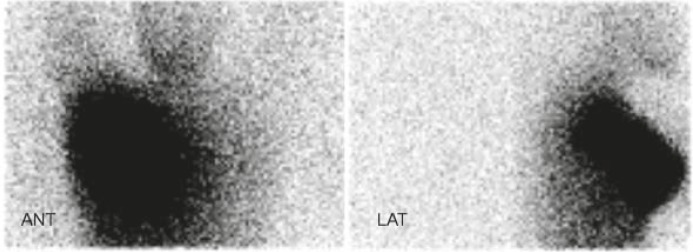
Anterior (left) and Lateral (right) scatter photon images of the patient shown on Figure 3. acquired 20 hours post-injection. Note poor quality of the images.

**FIGURE 5:**
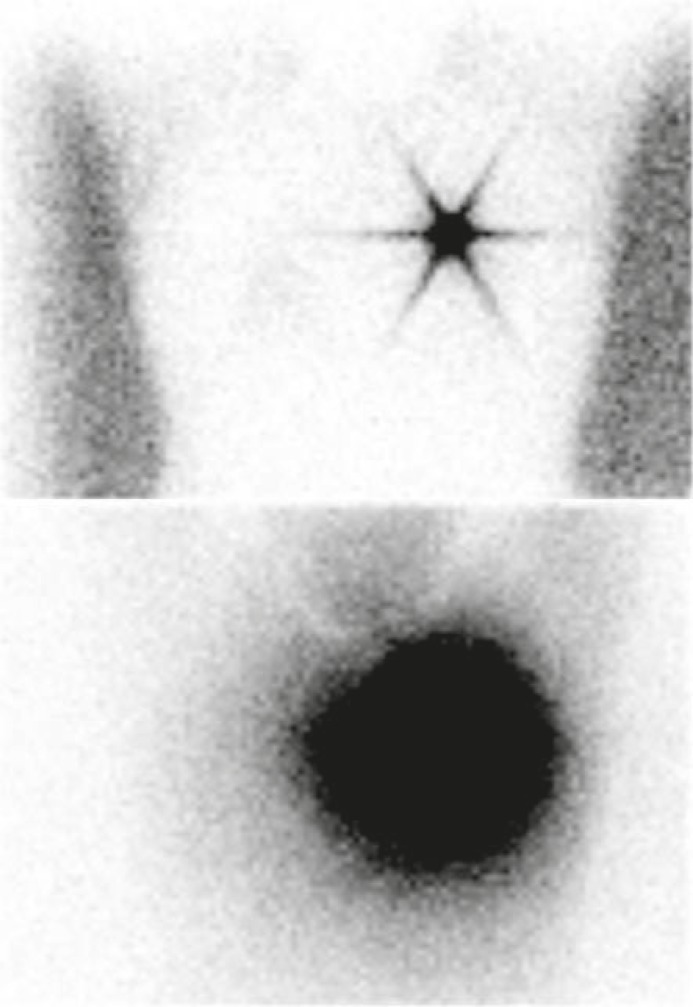
Body outline (top) and scatterogram (bottom) images of a patient. Note Concordance of body outlines in these two sets of image.

Characteristics of the patients are shown in Table 1. Lymphoscintigraphy images showed at least one axillary sentinel node in all patients (Figure 2). The early (30 minute image) scatterograms of the patients clearly showed the contour of the body (Figure 3). The 20 hour scatterograms were not as high quality as the corresponding early images and the body contours could not be clearly identified on these images (Figure 4). The constructed overlaid images showed the location of the axillary sentinel nodes and the body contours clearly for early scatterograms but not the delayed (20 hour) ones (Figure 1). In comparison with the corresponding outline images of the patients (using external flood source), no discordance was found regarding the body contour of the patients (Figure 5). No extra-axillary lymph drainage was noticed in our patients.

The processing of the images for the reconstruction of overlaid scatterograms took the mean time of 10±5 seconds.

## Discussion

As mentioned before several methods are in use for outlining the body contour during lymphoscintigraphy imaging. Providing the outline of the body can help the surgeons for better planning of the surgery with resulting the decrease in patients’ morbidity.^13^,^14^

The injection of small dose of Tc-99m-Pertechnetate to the patients can delineate the body outline as described by Klutmann *et al*. However, this method imposes an additional injection to the patients and also increases the radiation exposure.^11^

Manual outlining of the body using a Tc-99m point source and moving it around the patient’s edge is another way of body outlining. However, the images by this method are of low quality and still there is the additional radiation exposure to the patients and technologists.^4^

Using external gamma sources such as Co-57 and Gd-153 is another option. Despite using several methods for decreasing the radiation exposure^7^,^9^, Co-57 flood source poses additional radiation to the patients and personnel.^4^ Although additional radiation to the patients is lower with Gd-153 line sources^4^, acquiring body outline image with this method extends the time of examination which can interfere with the operation room scheduling. This is also true for all the methods mentioned above.^10^

The scattered photons of Tc-99m gamma rays are mainly the results of Compton scattering. As described by Fujii *et al.*, these photons occur everywhere in the body of the patients and imaging of these photons can show the contour of the body fairly well.^10^ We used the same method as described by Fujii *et al*. However, they used 111 MBq of the tracer in the peri-tumoral fashion.^10^ We use the intradermal injection of 18.5–37 MBq of the radiotracer for sentinel node mapping which produces less scattered photons compared to the Fujii *et al.* protocol. To obviate this problem we used the energy window of 60–120 keV for imaging of the scattered photons which is wider than the one used by Fujii *et al.* (70–110 keV).

Our study showed the same favourable results as the previous ones by a Fujii group. However, the images taken 20 hours post-injection were not of good quality at all which was due to scarcity of the scattered photon at the time of imaging.

One disadvantage of outlining the body with scattered photons is the non-homogenous distribution of these photons across the body of the patients. The scattered photons are densely distributed near the injection site and their density gradually decreases as the distance from the injection site increases.^12^ Because of this non-homogenous distribution of the scattered photons, simple summation of acquired images is not usually feasible since the sentinel node would be obscured and the exact location can not be identified. This is usually more problematic when the injection site is too close to the sentinel node location in the axilla.^12^ Fujii *et al.* developed a processing method to obviate this problem by dividing the primary photon image counts by the scattered photon image counts for each pixel after the addition of some constant counts to each pixel of the acquired image.^12^ These processing step was done for the compensation of count difference between two set of images. We used the NM-NM fusion workflow of E.CAM workstation to simply overlay the primary and scattered photon images after normalization of the images to the sentinel node counts on the primary photon images and the corresponding location in the axilla on the scattered photon ones. As both images were normalized to their corresponding optimized counts, the problem of the sentinel lymph node masking by the high activity of the injection site would be solved since the processing of each set of image was individualized and the optimal image for each patient could be reconstructed. Although Krynyckyi *et al.* considered this additional processing a drawback^7^, due to the simultaneous acquisition of both primary and scattered photons, there was no need for co-registration of the images and processing step could be done in a short time.

## Conclusions

Our study showed that imaging the scattered photons (scatterogram) during lymphoscintigrpahy could be a useful method for outlining the body contour of the patients and was feasible for the intradermal low dose injection of the radiotracers. This is especially true when the imaging is performed early after the injection of the tracer. This imaging method does not increase the radiation exposure of the patients or operators and does not extend the time of imaging either.

## Figures and Tables

**TABLE 1:** Characteristics of the patients

**Total number of patients**	**60**
Age	34±3
Tumor size	2.2±1.2
Tumor location	
Upper outer	25
Upper inner	15
Lower outer	7
Lower inner	8
Central	5
Histological type	
Invasive ductal	45
Invasive lobular	15
Biopsy type	
Excisional	20
Core needle	40
Time of surgery	
One-day protocol	22
Two-day protocol	38
